# Habitat Fragmentation Intensifies Trade-Offs between Biodiversity and Ecosystem Services in a Heathland Ecosystem in Southern England

**DOI:** 10.1371/journal.pone.0130004

**Published:** 2015-06-26

**Authors:** Justine E. Cordingley, Adrian C. Newton, Robert J. Rose, Ralph T. Clarke, James M. Bullock

**Affiliations:** 1 Centre for Conservation Ecology and Environmental Science, Faculty of Science and Technology, Bournemouth University, Dorset, United Kingdom; 2 Centre for Ecology and Hydrology, Bailrigg, Lancaster, United Kingdom; 3 Centre for Ecology and Hydrology, Wallingford, Oxfordshire, United Kingdom; Chinese Academy of Forestry, CHINA

## Abstract

While habitat fragmentation represents a major threat to global biodiversity, its impacts on provision of ecosystem services are largely unknown. We analysed biodiversity value and provision of multiple ecosystem services in 110 fragments of lowland heathland ecosystems in southern England, in which vegetation dynamics have been monitored for over 30 years. Decreasing fragment size was found to be associated with a decrease in biodiversity and recreational values, but an increase in relative carbon storage, aesthetic value and timber value. The trade-off between either biodiversity or recreational values with the other ecosystem services therefore became more pronounced as heathland size decreased. This was attributed to a higher rate of woody succession in smaller heathland fragments over the past three decades, and contrasting values of different successional vegetation types for biodiversity and provision of ecosystem services. These results suggest that habitat fragmentation can reduce the potential for developing “win win” solutions that contribute to biodiversity conservation while also supporting socio-economic development. Approaches to multi-purpose management of fragmented landscapes should therefore consider the potential trade-offs in ecosystem services and biodiversity associated with fragmentation, in order to make an effective contribution to sustainable development.

## Introduction

In recent years ecosystem services, or the benefits provided by ecosystems to people, have become a major international focus of research [1, 2, 3]. Notable research themes include the development of analytical frameworks and robust approaches to valuation [1, 4], analysis of the relationship between ecosystem services and biodiversity [5, 6], and the factors influencing the spatial and temporal dynamics of ecosystem services and their flows to beneficiaries [7, 8]. There is an increasing focus on how assessments of ecosystem services can be linked to socio-economic decision-making and associated policy, at both national and international scales [9, 10, 11]. Objectives relating to the provision of ecosystem services have been incorporated into international policy initiatives relating to biodiversity conservation and sustainable development, such as the Convention on Biological Diversity, Reducing Emissions from Deforestation and Forest Degradation (REDD+) [11] and the recently developed Intergovernmental Platform on Biodiversity and Ecosystem Services [12]. Ecosystem services are also becoming an increasingly tangible element of the global green economy, as a result of the widespread implementation of Payment for Ecosystem Service (PES) schemes [13, 14].

One of the principal outcomes of this recent research is a recognition of the importance of trade-offs between biodiversity and different ecosystem services [3, 15, 16, 17]. Trade-offs occur where increased provision of biodiversity or an ecosystem service is associated with reduced provision of other services [2]. Identification of such trade-offs is required to ensure that land use planning decisions can contribute effectively to addressing sustainability challenges [10, 11, 17]. Failure to recognise such conflicts can result in declines in biodiversity or provision of ecosystem services, with consequent impacts on human wellbeing [15]. Such trade-offs have been identified in a range of different environmental and socio-economic contexts. For example in Oregon, Nelson et al. [17] reported trade-offs between carbon sequestration and biodiversity conservation in different types of forested land, and a trade-off between both of these and agricultural production. Similarly in Hawaii, Goldstein et al. [10] identified trade-offs between carbon storage and water quality, and between these ecosystem services and agricultural production. In a Europe-wide study, Maes et al. [18] found that in general, biodiversity and ecosystem service supply were positively related, but spatial trade-offs were recorded among selected ecosystem services, in particular between crop production and regulating ecosystem services. Such trade-offs between agricultural production and biodiversity have been reported by a number of studies e.g. [8, 19, 20, 21], reflecting the impacts of conversion of ecosystems to agricultural land use. The causes of other trade-offs have rarely been examined, reflecting a lack of research on the drivers influencing ecosystem service relationships and their dynamics [15].

Here we examine the impact of a specific driver, namely habitat fragmentation, on trade-offs between biodiversity and ecosystem services. The process of habitat fragmentation refers to the division of continuous expanses of habitat into smaller discrete fragments, which are typically separated by some other type of land cover (such as agricultural land) that is relatively unsuitable for many species associated with the primary habitat. Habitat fragmentation is now widely recognised as a major cause of biodiversity loss [22, 23], and numerous studies have documented its impacts on the movement, distribution, reproduction and survival of individual species, as well as interactions between species and the structure of ecological communities [24, 25, 26]. Effects of fragmentation on ecosystem function, and the associated provision of ecosystem services, are far less well understood. According to theory, fragmentation could have significant impacts on ecosystem function at the local scale, although the effects may be delayed, and may be both complex and non-linear [26]. Relatively few field-based studies have investigated fragmentation impacts on ecosystem function. Examples are provided by Wardle et al. [27, 28], who found relationships between the area of islands in a Swedish archipelago and plant species composition, as well as ecosystem functions including plant litter decomposition, nitrogen mineralisation and uptake, and carbon partitioning. Further, Laurance et al. [29] documented a number of changes in ecosystem function in fragments of Amazonian forest surveyed over a 32-year period, including changes in hydrology, carbon storage and biochemical cycling.

On the basis of such evidence, we hypothesize that habitat fragmentation can affect both biodiversity and the provision of ecosystem services, and therefore the trade-offs between them. As far as we are aware, no previous investigation has examined the influence of habitat fragmentation on such trade-offs. We examined these impacts in fragments of lowland heathland, an ecosystem type that is widespread in north-west Europe and is recognised internationally as a priority for conservation, owing to its high biodiversity value [30, 31]. We analysed heathlands in the southern English county of Dorset, where their extent has decreased by about 85% over the past 200 years, leading to the creation of a large number of heathland fragments from what was once an almost continuous area [31, 32]. The vegetation dynamics of all remaining heathland fragments have been monitored in detail over the past three decades [32]. The availability of these long-term monitoring data for each of >100 heathland fragments provides a unique opportunity to examine the potential impacts of fragmentation on both biodiversity and ecosystem services. This investigation aimed to test the following hypotheses: (i) fragmentation has affected both biodiversity and the provision of ecosystem services in heathland fragments; (ii) contrasting responses of biodiversity and different ecosystem services to fragmentation has influenced trade-offs between them.

## Materials and Methods

### Ethical statement

Permission to conduct field surveys on each location was given by the individual landowners concerned, and by the regulatory authority (Natural England) in those situations where the field site was afforded protected status (i.e. Site of Special Scientific Interest). The research included interviews with recreational visitors to the study area. The research ethics committee of Bournemouth University specifically approved this study. Each voluntary respondent provided oral consent to participate in the research. The survey was conducted as a questionnaire-based interview with each respondent, and before the interview was conducted, respondents were asked whether or not they provided consent to participate. Only those who provided their consent participated in the survey. The responses were anonymised, and each respondent was made fully aware of how the data would be used, prior to being asked to provide their consent to participate in the survey. Written consent was not obtained as this was not requested by the University ethics committee, who stated that verbal consent would be sufficient. Verbal consent was recorded in the written questionnaire transcript. The ethics committee approved this consent procedure.

### Study area

The Dorset heathlands are situated in South West England (50°39’N, 2°5’W) and are located on Tertiary sands and gravels, associated with soils that are generally free-draining and acidic. The heathlands extend over an area of approximately 16,000 ha [32], and comprise a mosaic of different vegetation types, characterised by dwarf shrub communities dominated by members of the Ericaceae (e.g. *Calluna vulgaris*, *Erica* spp.), but also including areas of mire, grassland, scrub and woodland. Succession occurs on all sites with free-draining soils following a sequence from grassland to dwarf heath types, to scrub communities often dominated by *Ulex* spp., then to mixed woodland characterised by *Betula* spp., *Pinus* spp., *Quercus* spp. and *Salix* spp. [30, 31, 32]. Individual heathland fragments are managed for a variety of outcomes, including biodiversity conservation, recreation and timber production. The process of fragmentation and loss of the Dorset Heaths has been well documented, and has resulted from the increased expansion of agricultural and urban land, principally during the last century [31, 32]. There are currently 110 heathland fragments, with a mean area of 79 ha and a maximum area of 708 ha (see S1 Fig, S1 Table, S2 Table).

### The Dorset heathland survey

In 1978, a comprehensive vegetation survey was conducted on the Dorset heathlands and repeated, using the same approach, in the years 1987, 1996 and 2005. Detailed methods and results from the first three surveys have been published previously [32] but results from the 2005 survey, using the same methods, are presented here for the first time. For each survey, square plots of 4 ha (200 m x 200 m) were located based on the national Ordnance Survey mapping grid and were surveyed for the cover of all major vegetation types. These included four types associated with relatively dry soils (dry heath, grassland, scrub and woodland) and five additional types associated with relatively wet or poorly draining soils (brackish marsh, carr, humid heath, wet heath and mire). The other seven categories were bare ground, sand dunes, pools and ditches, sand and gravel, arable, urban and other land uses. The first survey in 1978 established 4 ha plots in all areas of the Dorset heathland region that contained some dwarf shrub heath, providing total coverage, and resulting in a total survey area of 3110 plots (12,440 ha). The same set of plots was resurveyed at each subsequent survey date. Within each plot, the cover of each vegetation type was recorded on a 3-point scale (1 = 1–10% cover; 2 = 10–50% cover; 3 = ≥50% cover). To calculate heathland areas, a land cover map was generated in ArcGIS 9.3 (ESRI, Redlands, California) from the 2005 heathland survey data. All 3110 squares in the survey were mapped. Squares were joined into heathland fragments using an 8 neighbour rule, i.e. either (i) horizontally- or vertically- adjacent or (ii) diagonally-adjacent squares containing heathland vegetation were joined. The area of each individual heathland fragment was calculated as the summed area of all squares in a fragment.

### Analysis of ecological succession

Linear regression analyses were performed in SPSS v.16.0 (SPSS Inc., Chicago) to test the relationship between fragment size and the extent of woody succession at the scale of the heathland fragment. Succession was measured as the proportional increase in area of woody vegetation (with a minimum increase of 1 ha) in each fragment, as recorded in the heathland survey. In addition to fragment area, the influence of the following predictor variables was tested, by deriving them from a national land cover map (LCM 2000; http://www.ceh.ac.uk/landcovermap2000.html) and the heathland survey data: (i) distance (m) to source populations of woody species, defined as the nearest area of woodland; (ii) the percentage of woodland cover within 500 m and 1000 m buffers around each individual heath fragment; and (iii) the percentage of urban land use within a 400 m buffer around each fragment. Both predictor and woody succession values were log transformed to achieve normality.

### Biodiversity value

The analysis of biodiversity value focused on species listed as being of conservation concern in the UK Biodiversity Action Plan (UKBAP; http://jncc.defra.gov.uk). Distribution records of BAP mammal, bird, reptile, amphibian, butterfly, vascular plant and bryophyte species were obtained from the Dorset Environmental Records Centre and the Amphibian and Reptile Conservation Trust. Records were filtered to include only those that: (a) were located within the extent of the Dorset heathlands, (b) were recorded at a resolution of 100 m or finer, (c) were collected between 2000 and 2010. Species of all habitat associations were included; in other words, the species considered were not limited to those specifically associated with or limited to heathland habitats. Distribution maps of each species were generated in ArcGIS, and overlaid on vegetation maps derived from the heathland survey data. Biodiversity value per unit area (species density) was calculated for each vegetation type within each individual heath fragment, by dividing the total number of species found within a vegetation type by the area of that vegetation type (following [21]). These values were averaged across all heaths to give a mean biodiversity value for each individual vegetation type.

### Ecosystem service assessment

Four ecosystem services were selected on the basis of their relatively high importance in heathlands: carbon storage, aesthetic value, recreation value and timber production. A value for each successional stage (grassland, dry heath, scrub and woodland) was obtained for each service, using the following methods.

#### Carbon storage

Carbon storage (t C ha^-1^) was assessed by measuring directly the amount of carbon in the following carbon pools: vegetation, soil (to 30 cm depth), roots, humus and dead organic matter. Measurements were conducted on ten heath fragments on sites that were selected using stratified random sampling methods, with random sampling applied within each vegetation cover type, and fragments stratified according to soil type. Vegetation and soil samples were taken from 0.01 ha circular plots in each vegetation type on each heath. Biomass and carbon content were determined using a FlashEA1112 Elemental Analyser (CE Instruments, Wigan, UK) in the laboratory. Detailed methods are provided in S1 Text.

#### Aesthetic value

Aesthetic value was measured by conducting a questionnaire survey of 200 heathland visitors distributed equally across the ten heaths used for carbon measurement, and eliciting preference values for each vegetation type using photo-realistic images. The aesthetic preference values were measured on a Likert scale (1–5) in a scoresheet, scoring how visually appealing the images were to heathland visitors. For further details, see S1 Text.

#### Recreational value

To examine the relationship between recreational value and vegetation type, the number of visitors to individual heaths was obtained from a questionnaire survey conducted by Liley et al. [33], which was sent to 5000 randomly selected postcodes from across the region. On the basis of the 1632 responses received, the number of visitors for each of 26 heaths was calculated, representing the heaths for which recreational visits were reported by respondents. The association between log-transformed values of vegetation cover and visitor number was then examined using Spearman’s rank correlation, using the proportion of each vegetation type in each heath calculated from the Dorset heathland survey data. To provide a measure of recreational value, vegetation types with significant negative correlations with visitor number were given a score of 1, those with significant positive correlations were accorded a score of 5, and those with no significant association were given a score of 3.

#### Timber value

Potential timber value was associated solely with broadleaved woodland. The extent of woodland cover on each heath was determined from the 2005 Dorset heathland survey data, supported by interpretation and digitisation of high resolution (25 cm) aerial photographs (Bluesky International Limited, Coalville, UK). Timber value was estimated following [21] using local yield data based on cumulative felling and local timber production values obtained from the Forestry Commission, UK.

#### Data analysis

Throughout, the analyses presented here focus on the four principal vegetation types (grassland, dry heath, scrub and woodland), which form a successional series on the drier heathland soils. To compare vegetation types with respect to biodiversity value, carbon storage and aesthetic value, the mean and median values per unit area were calculated for each vegetation type. Median values were then compared using Mann Whitney U tests. Analyses using the additional vegetation types present on heaths are presented in S2 Text.

The impact of habitat fragmentation was evaluated by calculating the relative value of biodiversity and each ecosystem service for each individual heath. This was achieved by first calculating the proportion of the area of each heath attributable to each vegetation type, using the 2005 heathland survey data. These proportional values were then multiplied by the biodiversity value and each individual ecosystem service value per unit area, for each vegetation type. Total values for each heathland fragment were calculated by summing these values across the four heathland vegetation types, at the level of each individual heath, following [21]. Heathland areas were calculated from the heathland survey data using ArcGIS. For analysing the effects of fragmentation, heathlands were grouped into three size categories: small (<50 ha), medium (50–300 ha) and large (>300 ha). Both mean and median values of biodiversity and each ecosystem service were calculated for each fragment size category, and were compared using either Mann-Whitney U-tests or t-tests, depending on the normality of the data. Normality was determined by performing a Kolmogorov-Smirnov test.

## Results

### Heathland succession

**Fig 1:**
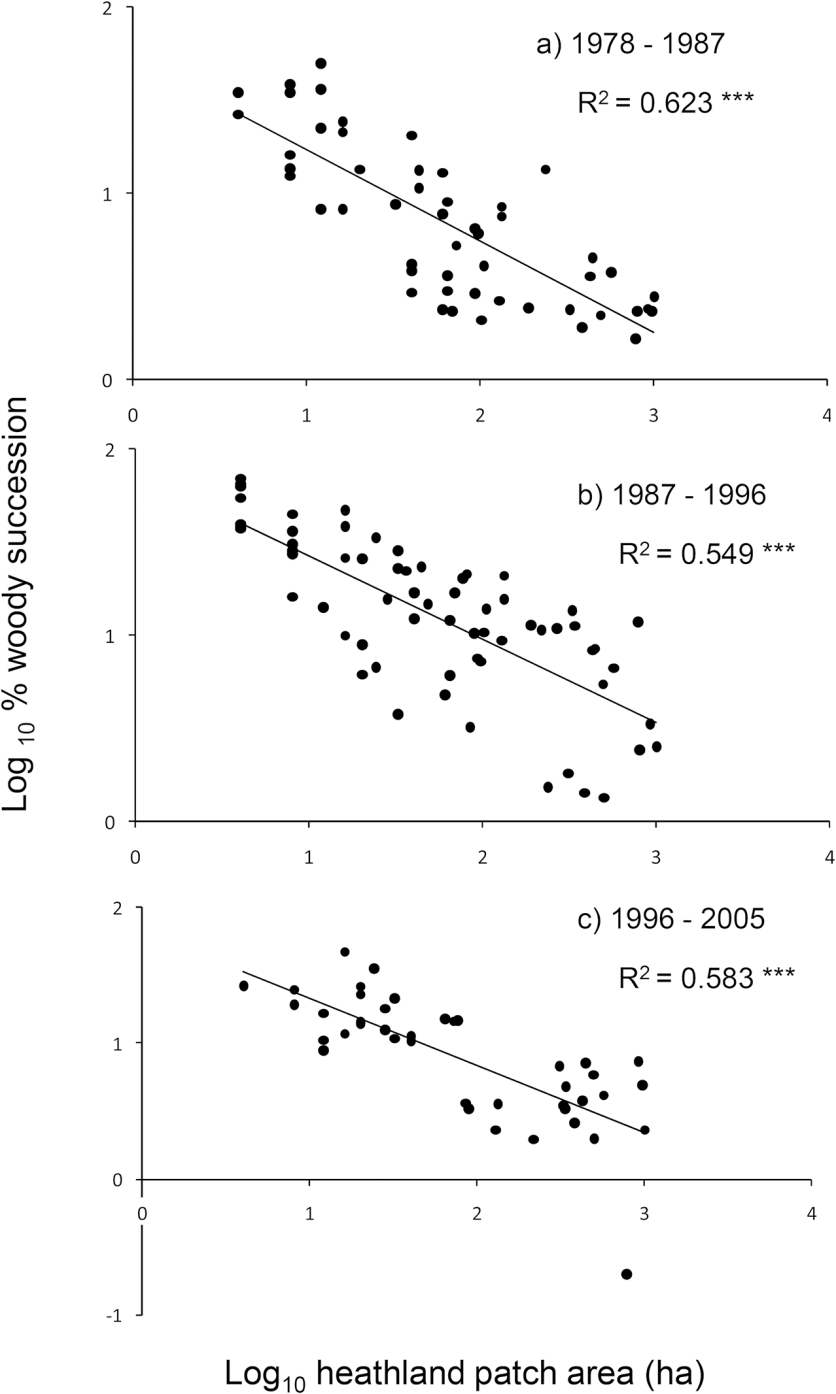
Relationship between heathland patch area (ha) and woody succession (both axes log_10_ transformed). Woody succession was measured as the proportional increase in area of woody vegetation (with a minimum increase of 1 ha) in each of 110 heathland fragments, derived from the Dorset heathland survey data, for the following intervals: a) 1978–87, b) 1987–96 and c) 1996–2005. Statistical significance of linear regressions indicated as *** P < 0.001, in each case. R^2^ indicates regression coefficients.

The rate of succession of heath to scrub and/or woodland was negatively related to heathland fragment size for each of the survey periods, with smaller heaths likely to undergo greater proportional increase in cover of woody vegetation types than larger heaths (Fig 1). None of the other factors tested were found to affect rate of succession, as no significant relationships (P >0.05, regression) were found between woody succession and distance to source populations, the amount of woodland surrounding a heath within 500 m and 1000 m, or the amount of urban development within 400 m of a heath.

### Biodiversity value

There was as expected a positive species–area relationship for all species of high conservation importance (Biodiversity Action Plan (BAP) species; see S2 Fig), and with a *z* value (slope of the log-log regression of the species-area curve) of 0.26. Overall, on the basis of the field observations that were analysed, the total number of BAP species associated with grassland (25) was lower than for dry heath, scrub or woodland (44, 46 and 45 species respectively). When the biodiversity data were corrected for area effects by calculating species density (i.e. number of BAP species per unit area), the biodiversity value of woodland was found to be lower than of the other vegetation types (P<0.05, t tests).

### Ecosystem services

**Fig 2:**
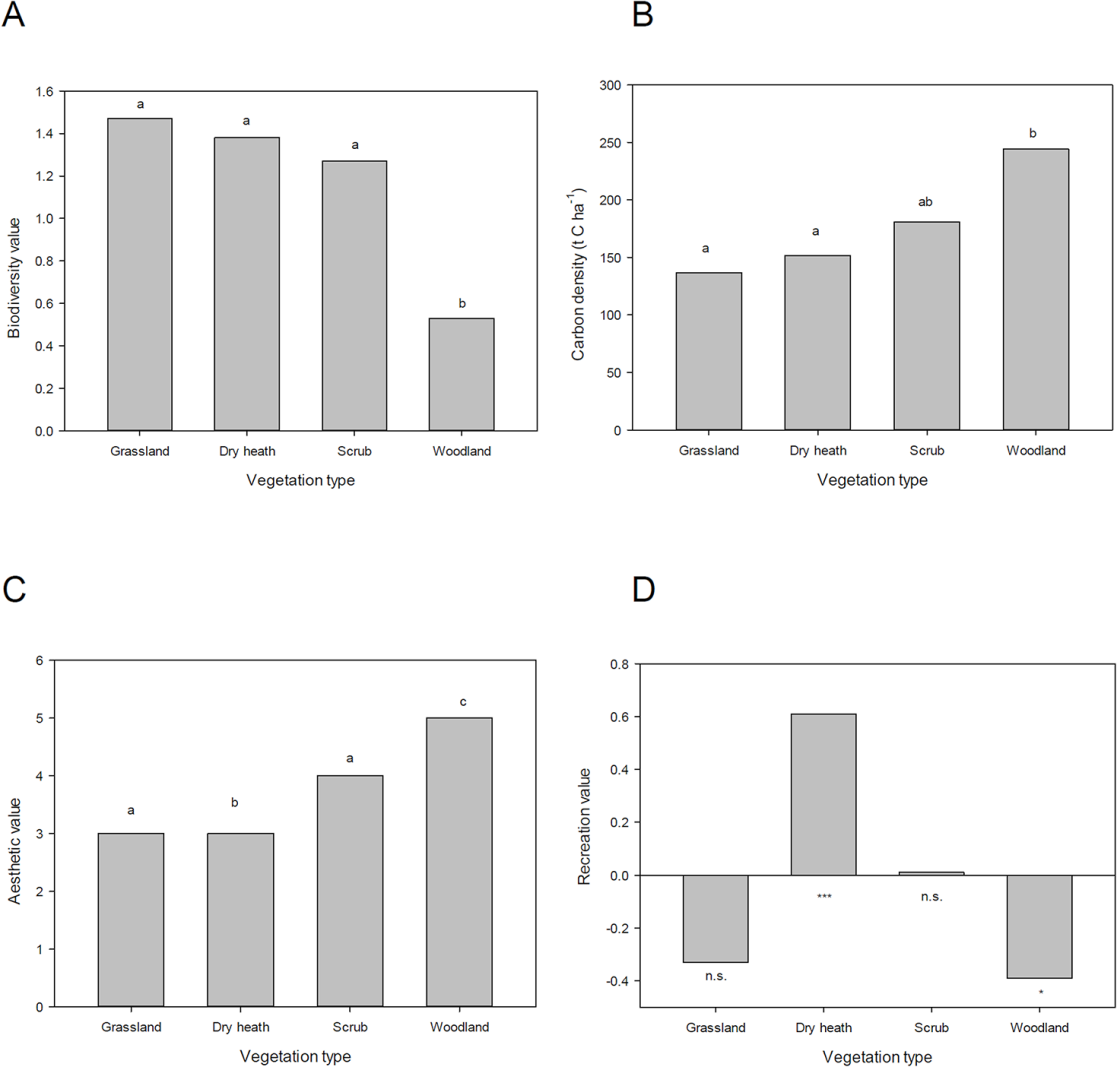
Relative value of four different vegetation types, situated along a successional gradient, for biodiversity and provision of ecosystem services. (a) Biodiversity value, presented as the mean number of records per hectare of species of conservation concern, (b) carbon density, presented as mean values of total (above- and below-ground) carbon storage per unit area (t C ha^-1^), (c) aesthetic value, presented as mean preference scores elicited from a visitor survey, and (d) recreational value, presented as the results of a correlation test between the number of heathland visitors and area of vegetation type (n.s., not significant; * P< 0.05, *** P< 0.001; y axis represents correlation coefficient). For (a)-(c), mean values grouped by the same letter are not significantly different (P< 0.05, Mann Whitney U test). For timber value, see text.

Provision of each ecosystem service differed between vegetation types (Fig 2). Carbon density was significantly higher in woodland than in dry heathland or grassland (P<0.05, t test). Similarly, aesthetic value of woodland was higher than of scrub, which was higher than of either grassland or dry heath (P<0.05, t test). Conversely, the number of heathland visitors was negatively related to woodland cover (P = 0.05, r = -0.39, Spearman rank correlation), whereas the relationship with dry heath was strongly positive (P <0.001, r = 0.61, Spearman rank correlation). No significant relationships were found between visitor numbers and the other vegetation types. Broadleaved woodland yields approximately 3 m^3^ ha^-1^ yr^-1^ of timber in this study area, on a 10-year cutting cycle. All other vegetation types were accorded a value of zero for timber production, as timber trees are either absent or occur at very low densities.

These results imply a number of trade-offs between biodiversity and ecosystem services, at the scale of individual vegetation types. Whereas carbon storage, aesthetic value and timber production were higher in woodland than in other vegetation types, such as dry heath, the converse pattern was observed for biodiversity value and for recreation.

### Habitat fragmentation

**Fig 3:**
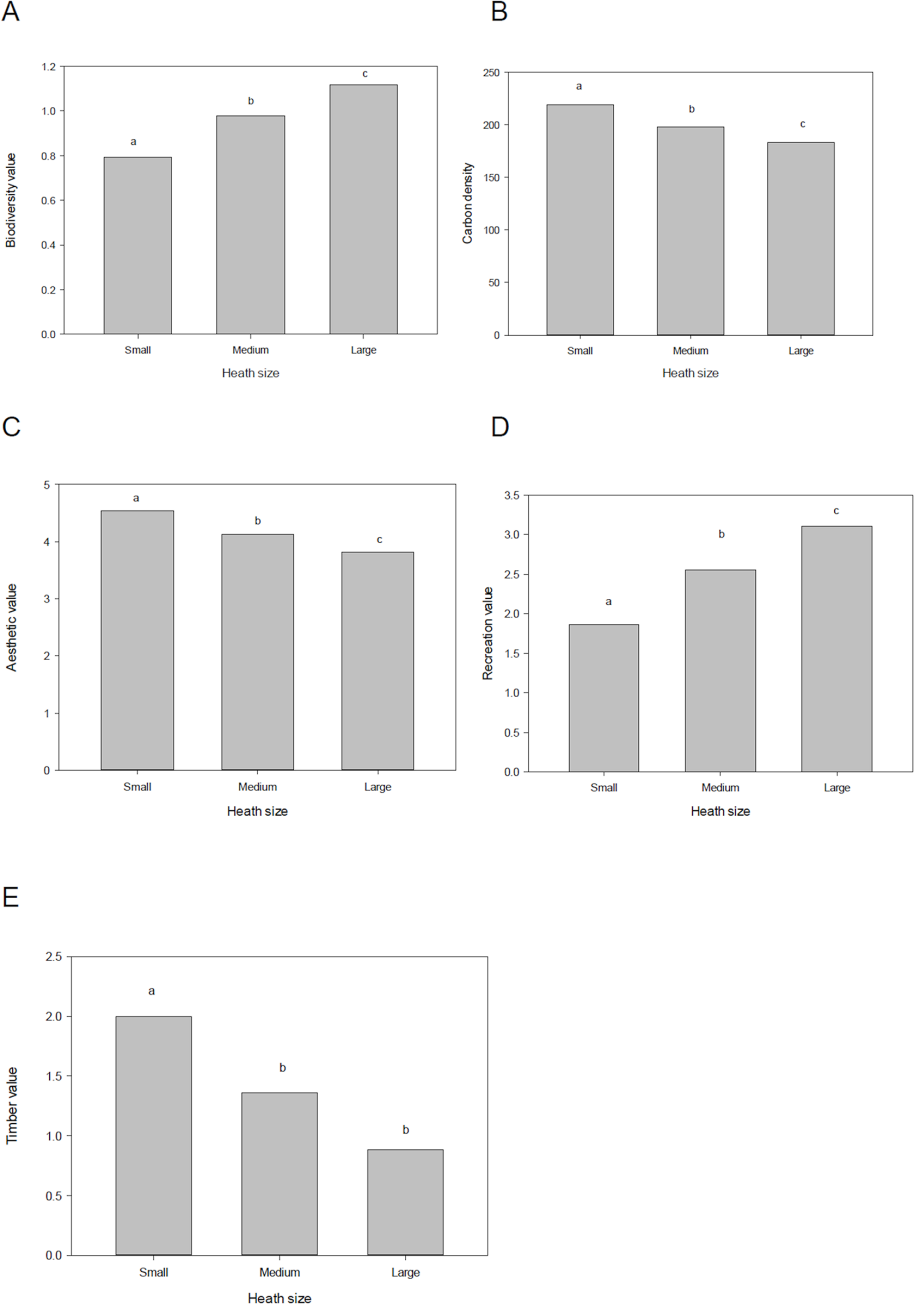
Relative value of different heathland fragment sizes for biodiversity and provision of ecosystem services. (a) Biodiversity value, (b) carbon density, (c) aesthetic value, (d) recreation value, and (e) timber value. Heathland fragments were divided into three size categories: small (<50 ha), medium (50–300 ha) and large (>300 ha). The values presented were produced by multiplying the proportion of heathland area comprising the different vegetation types, by the values per unit area of each type (see Fig 2). Values presented are means; those heathland sizes grouped by the same letter are not significantly different (P< 0.05, Mann Whitney U test conducted on median values).

Clear differences between heathland fragment size categories were observed for biodiversity value, and for each of the ecosystem services considered (Fig 3). In every case except timber, the ecosystem service values differed significantly between small, medium and large heath sizes (P <0.05, Mann Whitney U test). In the case of timber, the difference between large and medium-sized heath was not significant, but values of both were significantly lower than of small heaths.

Contrasting trends in the value of biodiversity and ecosystem services, per unit area of heathland (Fig 3), highlight the role of fragmentation in intensifying trade-offs. While decreasing fragment size was associated with a significant decrease in biodiversity value and a decrease in recreational value, the converse was observed for carbon storage, aesthetic value and timber value. In other words, the trade-off between either biodiversity or recreational value with the other ecosystem services became more pronounced as heathland size decreased. This was further illustrated by calculation of ratios between the mean values presented in Fig 3. When the mean value of carbon storage was divided by the mean value of biodiversity value, a ratio of 164 was obtained for large heaths. For small heaths, the value of this ratio was 276, an increase of 68%. Similarly, ratios increased by 68% for aesthetic value and 219% for timber value, when large and small heaths were compared. When ratios were calculated for recreational value rather than biodiversity, values for small heaths were more than double those of large heaths, for each of carbon storage, aesthetic and timber value.

## Discussion

This investigation has provided the first evidence that habitat fragmentation can intensify trade-offs between biodiversity and ecosystem services, and between different ecosystem services. In the heathland ecosystems studied here, a decrease in the size of habitat fragments was associated with a relative increase in carbon storage, aesthetic value and timber production, but a decrease in biodiversity value and recreational value. The negative impacts of habitat fragmentation on biodiversity are now widely recognised, and as a result, efforts to prevent or to reverse such fragmentation are becoming increasingly widespread [21, 34]. However, the potential impacts of fragmentation on provision of ecosystem services, and therefore human well-being, are largely unknown [35]. Our results highlight the importance of understanding the links between landscape structure and the provision of multiple ecosystem services, in order to develop effective approaches to the management of landscapes for biodiversity conservation and ecosystem services provision.

The results obtained here are attributable to an effect of habitat fragmentation on the process of ecological succession. Defined as the non-seasonal directional change in community composition over time, succession is widely recognised to be a major factor influencing the structure and composition of ecological communities [36]. The rate of succession is influenced by many processes, including the dispersal, colonisation, establishment and extirpation of species; interactions between species including competition, inhibition and facilitation; and both abiotic factors and disturbances [36, 37, 38]. Potentially, each of these processes could be influenced by habitat fragmentation. Similar negative interactions to that described here between habitat fragment size and rate of succession have been reported by some previous studies. For example Wardle et al. [28], working in an island archipelago in the boreal forest zone of Sweden, found that small islands were likely to undergo succession more rapidly, which they attributed to increased disturbance (fire caused by lightning strikes) on larger islands. Conversely, in experimentally fragmented agricultural fields, Cook et al. [39] reported more rapid woody succession in larger fragments, attributable to the presence of a larger number of suitable sites for seedling establishment. Such contrasting results highlight the difficulty of generalising about the impact of fragmentation on successional trajectories, particularly given the potential influence of stochastic events [40]. In the current investigation, the higher successional rate recorded in smaller fragments remains unexplained, although we hypothesize that this might be attributable to the proportionally higher length of edge habitat, which may have facilitated higher rates of colonisation by woody plants from neighbouring areas. This hypothesis requires further testing. Evidence from long-term plant metacommunity dynamics in this study area suggests that eutrophication and climate change may have accelerated woody plant succession in recent decades [41]; these processes could potentially have interacted with the size of heathland fragments to influence successional rates, but this also remains untested. A limitation of the current investigation is that analyses were largely limited to variation in patch size as a measure of fragmentation; potentially, variation in connectivity between habitat patches using measures other than those analysed here could also have influenced both the pattern of woody plant succession and the trade-offs observed. This limitation should be borne in mind when interpreting the results.

The influence of woody succession on ecosystem service and biodiversity trade-offs has been little studied. In a study of grasslands in New Zealand, Dickie et al. [42] found an increase in carbon pools with woody succession, but contrasting responses of species richness in different taxonomic groups. Based on these results, Dickie et al. [42] noted that management to maximize individual ecosystem services such as carbon sequestration may result in significant negative effects on biodiversity of some species groups. This has become an issue of major importance in the context of policy initiatives such as REDD+, which provides incentives to reduce emissions from forested lands and create financial value for carbon stored in forests [11, 43, 44]. The existence of trade-offs between carbon storage and biodiversity creates a risk that such interventions could result in a reduction in biodiversity value [11]. As noted by Wardle et al. [28], there have been few empirical tests of whether biodiversity and carbon sequestration are influenced by the same factors, and whether management for one of these variables can also maximise the other. In tropical forests, Martin et al. [43] have recently shown that while biodiversity and carbon storage may be positively related, they are both dynamic over time, and differ in their rate of recovery during secondary succession following disturbance. While a number of other studies have suggested that biodiversity and carbon storage can be positively related [44], others have suggested that trade-offs can occur between them, particularly at local to regional scales [18], as recorded here.

The current results suggest that trade-offs between carbon storage and biodiversity are more likely when early successional habitats are associated with relatively high biodiversity value, and that such trade-offs can be intensified by habitat fragmentation if smaller fragments are associated with higher rates of succession. It is unclear how widespread such interactions might be, although many early-successional plant communities in north-western Europe are of relatively high biodiversity value, including semi-natural grasslands and shrublands. Other ecosystem services that may be influenced by fragmentation include pollination, seed dispersal and pest regulation, although evidence for this is limited [35]. Information on cultural services is particularly lacking. In the current study, a trade-off was recorded between two cultural services, namely recreational and aesthetic value. In many countries, outdoor recreation is a major leisure activity enjoyed by large parts of the population [45]. In England, there are around 2,858 million outdoor recreational visits made every year, with at least 59% of the population visiting the countryside within a period of a year [46]. The high recreational use of heathland reflects a preference for the relatively open heath vegetation for walking and associated forms of recreation, particularly the exercising of dogs [33]. The trade-off recorded here suggests people do not choose recreation opportunities based solely on aesthetic values, but on criteria such as convenience and accessibility, a finding supported by other investigations [33, 45]. The trade-off documented here may therefore be widespread, and may be influenced by habitat fragmentation when contrasting recreational and aesthetic values are associated with different stages of ecological succession, as recorded here.

Bennett et al. [15] highlighted the importance of understanding trade-offs and synergies between ecosystem services so that relationships between them can be effectively managed, thereby strengthening ecosystem resilience, enhancing the provision of multiple services, and avoiding catastrophic shifts in ecosystem service provision. Explicit recognition of such trade-offs is also crucial for evaluating the potential of ‘win-win’ solutions that will contribute to biodiversity conservation while also supporting social and economic development [17, 47, 48]. The intensification of trade-offs with declining fragment size recorded here suggests that such ‘win-win’ solutions might be more difficult to achieve in areas subjected to habitat fragmentation. In such circumstances, a regional-scale approach to land management planning may be required, which explicitly recognises the contrasting values of differently sized fragments and the trade-offs associated with them. The current results show that small fragments of relatively low biodiversity value could potentially be of relatively high value for ecosystem service provision, and consequently for human well-being, and may therefore require explicit consideration in management planning and policy development. These results also have implications for the development of ecological networks through ecological restoration, an approach that is being widely implemented [34]. While the development of ecological networks can benefit biodiversity by increasing the connectivity between habitat fragments, few studies have examined their potential impact on ecosystem services [21]. Our results suggest that development of an ecological network and the consequent increase in fragment size could potentially reduce the relative provision of some ecosystem services, and thereby reduce the overall cost-effectiveness of the network. Such observations highlight the need to consider the potential impacts of fragmentation on ecosystem service provision, as well as biodiversity, when developing multi-purpose approaches to landscape management.

## Supporting Information

S1 Fig (a) The location and current extent of the Dorset heathlands, UK and (b) the 3110 4 ha squares of the Dorset heathland survey surveyed in 1978, 1987, 1996 and 2005. (PDF)

S2 Fig Species-area relationship of number of species of conservation concern (BAP species) (log_10_) per heath plotted against heathland fragment area (ha) (log_10_). Species number was determined from local survey data recorded on the Dorset heathlands between 2000 and 2010. Heathland area (ha) was calculated from a 2005 digitised map of the Dorset heathlands. Species records were mapped onto the digitised heathland map to determine the species-area relationship (*z* = 0.265, R^2^ = 0.628).
(DOC)

S1 Table Total area (ha) of heathland, associated vegetation types and other categories recorded in surveys in 1978, 1987, 1996 and 2005 across the original 3110 squares of the Dorset heathland survey. The % area change shows the % increase or decrease a category underwent between surveys. The ‘other’ category includes sand dunes with heather, pools and ditches, sand and gravel, arable, wet heath and mire, urban and other land use. Arable, urban and other land uses were only recorded specifically in 1996 and 2005.
(DOC)

S2 Table Fragmentation metrics for the Dorset heathlands over four surveys calculated using FRAGSTATS [49]. Heathland survey squares were joined into patches if they contained some heathland (dry heath, humid heath, wet heath and mire) based on an 8 cell neighbour rule. Area and distance values grouped by different letters are significantly different within each column (Mann-Whitney U test P < 0.05).
(DOC)

S1 Text Methods for assessing provision of ecosystem services. (DOC)

S2 Text Analysis of vegetation types. (DOC)
