# Supplementary material for: Habitat Fragmentation Intensifies Trade-Offs between Biodiversity and Ecosystem Services in a Heathland Ecosystem in Southern England
Source: PLoS One. 2015 Jun 26;10(6):e0130004. doi: 10.1371/journal.pone.0130004 (PMC4483160; doi:10.1371/journal.pone.0130004)

Figure S1. Map of Dorset heathland.

(a) The location and current extent of the Dorset heathlands, UK and (b) the 3110 4 ha squares of the Dorset heathland survey surveyed in 1978, 1987, 1996 and 2005.

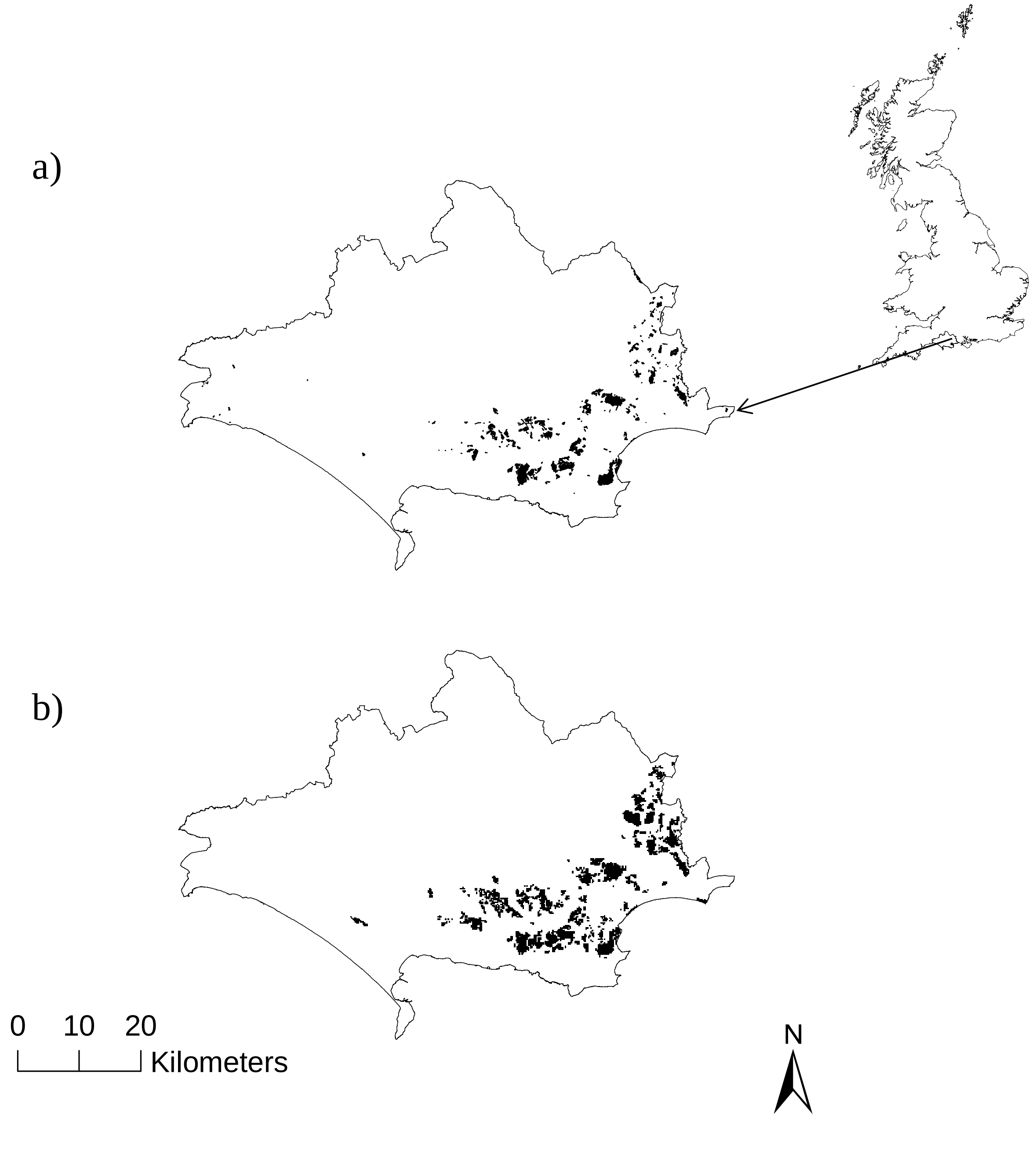

Supplement: S1 Fig — (PDF) [file pone.0130004.s001.pdf]
