# Supplementary material for: Habitat Fragmentation Intensifies Trade-Offs between Biodiversity and Ecosystem Services in a Heathland Ecosystem in Southern England
Source: PLoS One. 2015 Jun 26;10(6):e0130004. doi: 10.1371/journal.pone.0130004 (PMC4483160; doi:10.1371/journal.pone.0130004)
Supplement: S2 Fig — Species number was determined from local survey data recorded on the Dorset heathlands between 2000 and 2010. Heathland area (ha) was calculated from a 2005 digitised map of the Dorset heathlands. Species records were mapped onto the digitised heathland map to determine the species-area relationship (z = 0.265, R2 = 0.628). (DOC) [file pone.0130004.s002.doc]

**Figure S2.** Species-area relationship of number of species of conservation concern (BAP species) (log_10_) per heath plotted against heathland fragment area (ha) (log_10_). Species number was determined from local survey data recorded on the Dorset heathlands between 2000 and 2010. Heathland area (ha) was calculated from a 2005 digitised map of the Dorset heathlands. Species records were mapped onto the digitised heathland map to determine the species-area relationship (*z* = 0.265, R² = 0.628).
