# Supplementary material for: Habitat Fragmentation Intensifies Trade-Offs between Biodiversity and Ecosystem Services in a Heathland Ecosystem in Southern England
Source: PLoS One. 2015 Jun 26;10(6):e0130004. doi: 10.1371/journal.pone.0130004 (PMC4483160; doi:10.1371/journal.pone.0130004)
Supplement: S1 Table — The % area change shows the % increase or decrease a category underwent between surveys. The ‘other’ category includes sand dunes with heather, pools and ditches, sand and gravel, arable, wet heath and mire, urban and other land use. Arable, urban and other land uses were only recorded specifically in 1996 and 2005. (DOC) [file pone.0130004.s003.doc]

**S1 Table.** Total area (ha) of heathland, associated vegetation types and other categories recorded in surveys in 1978, 1987, 1996 and 2005 across the original 3110 squares of the Dorset heathland survey. The % area change shows the % increase or decrease a category underwent between surveys. The ‘other’ category includes sand dunes with heather, pools and ditches, sand and gravel, arable, wet heath and mire, urban and other land use. Arable, urban and other land uses were only recorded specifically in 1996 and 2005.

| Vegetation cover type | 1978 area (ha) | 1987  area (ha) | 1996  area (ha) | 2005  area (ha) | t78-87  area change (%) | t87-96  area change (%) | t96-05  area change (%) | t78-05  area change (%) |
| --- | --- | --- | --- | --- | --- | --- | --- | --- |
|  |  |  |  |  |  |  |  |  |
| Dry heath | 2554 | 2016 | 2072 | 1872 | *-21* | *3* | *-10* | *-27* |
| Scrub | 1018 | 1178 | 1405 | 1488 | *16* | *19* | *6* | *46* |
| Woodland | 1830 | 1942 | 2433 | 2651 | *6* | *25* | *9* | *45* |
| Grassland | 43 | 103 | 229 | 783 | *140* | *122* | *242* | *1721* |
| Other vegetation | 3152 | 3331 | 2894 | 125 | *6* | *-13* | *-96* | *-96* |
|  |  |  |  |  |  |  |  |  |
| **Total :** | **8597** | **8570** | **9033** | **6919** | ***-0.3*** | ***5*** | ***-23*** | ***-20*** |
|  | | |  |  |  |  |  |  |
