# Supplementary material for: Habitat Fragmentation Intensifies Trade-Offs between Biodiversity and Ecosystem Services in a Heathland Ecosystem in Southern England
Source: PLoS One. 2015 Jun 26;10(6):e0130004. doi: 10.1371/journal.pone.0130004 (PMC4483160; doi:10.1371/journal.pone.0130004)
Supplement: S2 Table — Heathland survey squares were joined into patches if they contained some heathland (dry heath, humid heath, wet heath and mire) based on an 8 cell neighbour rule. Area and distance values grouped by different letters are significantly different within each column (Mann-Whitney U test P < 0.05). (DOC) [file pone.0130004.s004.doc]

**S2 Table.** Fragmentation metrics for the Dorset heathlands over four surveys calculated using FRAGSTATS (McGarigal et al. 2002). Heathland survey squares were joined into patches if they contained some heathland (dry heath, humid heath, wet heath and mire) based on an 8 cell neighbour rule. Area and distance values grouped by different letters are significantly different within each column (Mann-Whitney U test P < 0.05).

|  | Total number of heath fragments | Total number of heath fragments under 10 ha | Mean area  (ha) | Maximum area  (ha) | Median area  (ha) | Mean distance to nearest heath  (km) | Median distance to nearest heath  (km) |
| --- | --- | --- | --- | --- | --- | --- | --- |
|  |  |  |  |  |  |  |  |
| 1978 | 112 | 31 | 111 ^a^ | 992 ^a^ | 30 ^a^ | 0.69 ^a^ | 0.40 ^a,c^ |
| 1987 | 130 | 45 | 90 ^a^ | 992 ^a^ | 22 ^a^ | 0.63^a^ | 0.40 ^a^ |
| 1996 | 130 | 47 | 78 ^a^ | 820 ^a^ | 18 ^a^ | 0.61 ^a^ | 0.45 ^b,c^ |
| 2005 | 110 | 35 | 79 ^a^ | 708 ^a^ | 20 ^a^ | 0.63 ^a^ | 0.45 ^c^ |
|  |  |  |  |  |  |  |  |

**Reference**

McGarigal K, Cushman SA, Neel MC, Ene E (2002) Fragstats: Spatial Pattern Analysis Program for Categorical Maps. University of Massachusetts, Landscape Ecology Program. Website:

[www.umass.edu/landeco/research/fragstats/fragstats.html](http://www.umass.edu/landeco/research/fragstats/fragstats.html)
