# Supplementary material for: Habitat Fragmentation Intensifies Trade-Offs between Biodiversity and Ecosystem Services in a Heathland Ecosystem in Southern England
Source: PLoS One. 2015 Jun 26;10(6):e0130004. doi: 10.1371/journal.pone.0130004 (PMC4483160; doi:10.1371/journal.pone.0130004)
Supplement: S1 Text — (DOC) [file pone.0130004.s005.doc]

**S1 Text. Methods for assessing provision of ecosystem services**

**Carbon stocks**

Carbon storage values were quantified for dry heath (pioneer, building and degenerate life-cycle stages), humid heath, grassland, scrub and woodland. Ten heathlands were identified which contained each of these vegetation types. Within each heathland, sites for each major vegetation category (*n* = 70) were chosen using stratified random sampling using Hawth's Analysis Tools for ArcGIS 9.3. A 100 m edge buffer was established around each heath and no points were placed within this buffer zone. Field work was carried out between July and October 2010 (for above-ground carbon stocks) and July and October 2011 (for soil carbon stocks). One circular plot of 5.6 m radius (0.01 ha) was established at each site. All vascular plant species within plots were identified by species and a note made of moss and lichen presence. Within each plot, ground cover biomass was measured by harvesting all vegetation in four 0.25 m^2^ quadrats following percentage cover estimates (visual and pin drop method) for each species. Biomass was sorted into species and then into the component parts (leaves and branches), from which samples were taken and weighed and then dried at 60°C for 48 hrs. Samples were bulked for each species (and component parts) for each site and sub-samples were ground using a ball-mill and analysed for carbon and nitrogen content using a FlashEA1112 Elemental Analyser (CE Instruments, Wigan, UK). For tree species, individual trees were defined as seedlings (a living stem less than 50 cm tall), saplings (a living stem greater than 50 cm tall and with a diameter at breast height (dbh) less than 7 cm) or trees (a living stem with a dbh greater than 7 cm). Total carbon was assigned to seedlings and saplings based on height. Biomass of trees was estimated by direct measurement of the diameter and heights of each tree, in each site. Biomass was calculated for the stem and crown of trees in each plot following the procedure, which includes the use of allometric equations, used by the UK Forestry Commission (Jenkins et al. 2011). Carbon content was assumed to be 50% of tree biomass.

In relation to soil carbon, clearly defined soil horizons were not present but the litter layer, humus layer and soil layer were easily distinguishable. Two volumetric pits were dug to 50 cm at 2.5 m from the centre of the plot. Soil was extracted separately for depths of 0-5, 5-10, 5-30 and 30-50 cm after removing the humus and litter layers. A corer was used to core soil from 50-70 cm and bulk density for this depth assumed to be the same as the 30-50 cm depth. Soil was stored at 4°C in the field, air-dried and then processed (passed through a 10 mm and then 2 mm sieve where stones and organic material were removed, weighed and the volume measured) to estimate bulk density. For each site, sieved soil was pooled from the two volumetric pits for each depth increment, ball-milled and analysed for carbon and nitrogen using a FlashEA1112 Elemental Analyser.

Root biomass for individual trees was estimated using allometric equations used by the UK Forestry Commission and carbon was assumed to be 50% of root biomass (Jenkins et al. 2011). In addition, for each site root biomass was measured from roots extracted in samples from the volumetric pits (0-50 cm). Root biomass ground vegetation was estimated by hand-picking roots from 10 mm and 2 mm sieves which were then washed with de-ionised water to remove all soil, pebbles and debris. Roots were dried at 60°C for 48 hrs. or until dry and weighed for biomass. Roots over 10 mm were ground and analysed for carbon and nitrogen using a FlashEA1112 Elemental Analyser and the carbon value applied to the root biomass.

Within each site, the soil humus layer was sampled from four locations using a 300 cm^2^ frame. Live plant material was removed from inside the frame and then a knife was used to cut out the humus layer from inside the frame, down to the surface of the soil mineral layer. Mean humus depth was measured three times along the frame for each location. Humus samples were dried at 60°C for 48 hrs. Humus samples were sieved and stones were removed by hand. Samples were pooled for each site and a sub-sample was ground using a ball-mill and analysed for carbon and nitrogen content using a FlashEA1112 Elemental Analyser.

Dead organic matter consisted of leaf litter and standing dead wood. For standing dead trees, measurements were made of dbh, height and the decomposition state of each tree. Dbh and height were measured using tapes. Biomass and carbon content was calculated in the same way as for live trees. Volume was converted to biomass using wood density factors from Sandström et al. (2007). Carbon was assumed to be 50% of total biomass.

Within each site, the leaf litter layer was sampled from the same four locations as the humus using a 300 cm^2^ frame. All leaf litter was removed from inside the frame, dried at 60°C for 48 hrs. (or until dry), sieved and stones were removed by hand. Samples were pooled for each site and a sub-sample was ground using a ball-mill and analysed for carbon and nitrogen content using a FlashEA1112 Elemental Analyser.

**Aesthetic value**

A questionnaire survey of heathland visitors was conducted to collect information on (i) aesthetic values for images of individual heathland vegetation types, (ii) demographic of respondents, (iii) heathland use by respondents. For (i), images were printed as high-quality photographs (12.2 cm x 8.1 cm) and presented in a random order on two sides of a soft board (41.9 cm x 30 cm) which the respondent could choose to hold. Respondents were asked to rate each image on a 5-step scale of regarding how aesthetically appealing they considered it. The photo-realistic images were created to represent a range of successive heathland vegetation cover types. Photos of different heathland communities were taken over five days in August 2011, using a Nikon D200 with a wide angle 20 mm lens. A range of photographs were taken of different heathland cover types on six heaths. A single base photograph representing a ‘typical’ heathland scene was then chosen from this set of photographs. This base photograph was then altered using photo-editing in Adobe Photoshop CS 5.1 (Adobe Systems Europe Ltd, Maidenhead, UK) to produce single images of individual heathland cover types. Altering only the vegetation cover types within each image ensured that any differences in preference values for different heathland communities could be assumed to be based on the difference in the vegetation itself rather than any external features. Skylines were standardised. Images of different heathland communities were created by laying clipped images of each individual vegetation community on top of the base image, including: (a) grassland; (b) mire; (c) humid/wet heath; (d) dry heath; (e) dry heath in flower; (f) a close up view of scrub; (g) scrub; (h) a distant view of scrub; (i) a distant view of woodland and (j) mixed mature woodland. Questionnaire interviews were conducted across ten heathlands in Dorset in July and August 2012. Each heathland was visited between 7:30 am and 2:30 pm and between 5:00 pm and 7:30 pm until 20 respondents had been surveyed on each heath. Anonymity was guaranteed to study participants.

**References**

Jenkins TAR et al. (2011) *FC Woodland Carbon Code: Carbon Assessment Protocol*. Forestry Commission, Edinburgh, UK.

McGarigal K, Cushman SA, Neel MC, Ene E. (2002) *FRAGSTATS. Spatial pattern analysis program for categorical maps. Computer software program produced by the authors at the University of Massachusetts, Amherst.*

Sandström F, Petersson H, Kruys N, Ståhl G (2007) Biomass conversion factors (density and carbon concentration) by decay classes for dead wood of *Pinus sylvestris*, *Picea abies* and *Betula* spp. in boreal forests of Sweden. *For Ecol Manage* 243(1): 19–27.
