# Supplementary material for: Habitat Fragmentation Intensifies Trade-Offs between Biodiversity and Ecosystem Services in a Heathland Ecosystem in Southern England
Source: PLoS One. 2015 Jun 26;10(6):e0130004. doi: 10.1371/journal.pone.0130004 (PMC4483160; doi:10.1371/journal.pone.0130004)
Supplement: S2 Text — (DOC) [file pone.0130004.s006.doc]

**S2 Text. Analysis of vegetation types**

In the main text, the comparison of heathland fragment sizes in relation to biodiversity value and provision of ecosystem services focused on the four heathland vegetation types that form a successional series on relatively dry soils (i.e. grassland, dry heath, scrub and woodland). This was achieved by first calculating the proportion of the area of each heath attributable to each vegetation type, using the 2005 heathland survey data. These proportional values were then multiplied by the biodiversity value and each individual ecosystem service value per unit area, for each vegetation type. Total values for each heathland fragment were calculated by summing these values across the four selected vegetation types, at the level of each individual heath. These analyses were repeated, using proportions of all of the vegetation types present within each individual heath, rather than solely the four associated with dry soils. Results were broadly similar to those presented in the main text (Table S2), with trade-offs recorded between biodiversity and recreational value versus carbon storage and timber value, intensified by fragment size. The only exception was aesthetic value, which did not show a significant difference between heathland fragment sizes in this analysis.

**Table S2.** Effect of heathland size on biodiversity value and provision of ecosystem services, using proportional values based on all vegetation types present. Values presented are means + SE. Statistical analysis: * P ≤ 0.05, ** P ≤ 0.01, *** P ≤ 0.001. T test performed on means, Mann Whitney U test performed on median values, depending on normality of data.

|  | **Heathland fragment size** | | | **Statistical analysis** |
| --- | --- | --- | --- | --- |
|  | Small | Medium | Large |  |
| Biodiversity value  (no. BAP species ha^-1^) | 0.57+ 0.025 | 0.66 + 0.03 | 0.73 + 0.03 | Small v medium *  Small v large **  Medium v large n.s.  (T test) |
| Aesthetic value | 3.17 + 0.12 | 2.73 + 0.16 | 2.41 + 0.19 | All comparisons n.s. (Mann Whitney U test) |
| Carbon storage  (t C ha^-1^) | 152.7 + 6.0 | 131.3 + 7.6 | 115.9 + 8.8 | Small v medium *  Small v large **  Medium v large n.s.  (T test) |
| Recreation value | 1.36 + 0.07 | 1.75 + 0.13 | 2.03 + 0.06 | Small v medium *  Small v large *  Medium v large *  (Mann Whitney U test) |
| Timber value (m^3^ ha^-1^ yr^-1^) | 1.35 + 0.08 | 0.89 + 0.13 | 0.51 + 0.10 | Small v medium *  Small v large *  Medium v large n.s.  (Mann Whitney U test) |
